# Author Correction: Robust and tunable signal processing in mammalian cells via engineered covalent modification cycles

**DOI:** 10.1038/s41467-023-40559-5

**Published:** 2023-08-08

**Authors:** Ross D. Jones, Yili Qian, Katherine Ilia, Benjamin Wang, Michael T. Laub, Domitilla Del Vecchio, Ron Weiss

**Affiliations:** 1 Department of Biological Engineering, Massachusetts Institute of Technology, Cambridge, MA 02139 USA; 2 Synthetic Biology Center, Massachusetts Institute of Technology, Cambridge, MA 02139 USA; 3 Department of Mechanical Engineering, Massachusetts Institute of Technology, Cambridge, MA 02139 USA; 4 Department of Biology, Massachusetts Institute of Technology, Cambridge, MA 02139 USA; 5 Howard Hughes Medical Institute, Massachusetts Institute of Technology, Cambridge, MA 02139 USA; 6 Electrical Engineering and Computer Science Department, Massachusetts Institute of Technology, Cambridge, MA 02139 USA

**Keywords:** Synthetic biology, Signal processing, Control theory, Genetic circuit engineering, Phosphorylation

Correction to: *Nature Communications* 10.1038/s41467-022-29338-w, published online 31 March 2022

In the original version of this Article, the directional arrows in figure panels Fig. 2e and Fig. 2f indicating the transition between OmpR tagged with VP64 and phosphorylated OmpR tagged with VP64 were reversed. The correct figure is presented below. This has been corrected in the HTML and PDF version of the paper. In addition, the caption for Supplementary Fig. 17b “Red symbols correspond to the calculations shown in Panel (a) and Fig. 5d (threshold = 105 MEFLs Output)” was incorrectly presented as “Red symbols correspond to the calculations shown in Fig. 2c (threshold = 105 MEFLs Output – also shown drawn on plots in (a))”. The corrected Supplementary Information file is appended below.


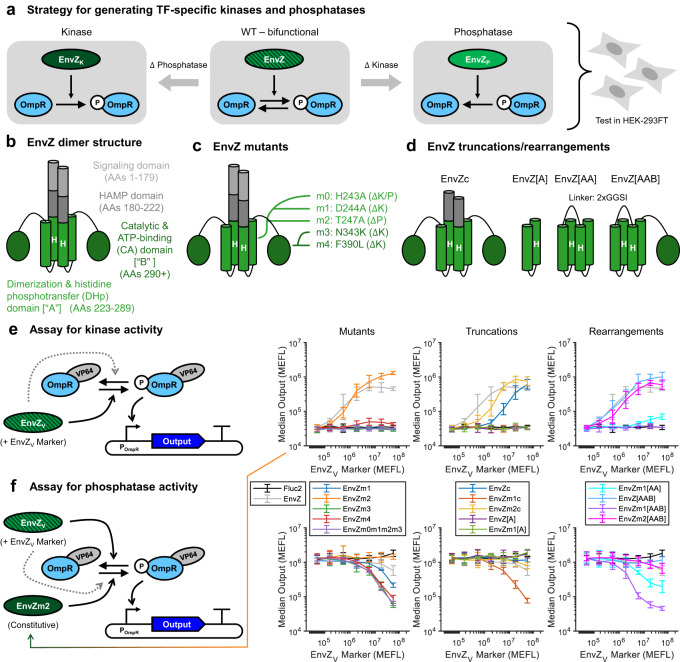


### Supplementary information

Updated Supplementary Information

