# Supplementary material for: Robust and tunable signal processing in mammalian cells via engineered covalent modification cycles
Source: Nat Commun. 2022 Mar 31;13:1720. doi: 10.1038/s41467-022-29338-w (PMC8971529; doi:10.1038/s41467-022-29338-w)
Supplement: Supplementary file 5 — Description of Additional Supplementary Files [file 41467_2022_29338_MOESM5_ESM.pdf]

Title: Supplementary Data 1
Description: Contains plasmid sequences transfection tables.
